# Parkin Coordinates Platelet Stress Response in Diabetes Mellitus: A Big Role in a Small Cell

**DOI:** 10.3390/ijms21165869

**Published:** 2020-08-15

**Authors:** Seung Hee Lee, Jing Du, John Hwa, Won-Ho Kim

**Affiliations:** 1 Division of Cardiovascular Diseases, Center for Biomedical Sciences, National Institute of Health, Cheongju-si 28159, Chungbuk, Korea; jhkwh@nih.go.kr; 2 Yale Cardiovascular Research Center, Section of Cardiovascular Medicine, Department of Internal Medicine, Yale University School of Medicine, New Haven, CT 06511, USA; Jing.du@yale.edu (J.D.); john.hwa@yale.edu (J.H.)

**Keywords:** platelet, diabetes, Parkin

## Abstract

Increased platelet activation and apoptosis are characteristic of diabetic (DM) platelets, where a Parkin-dependent mitophagy serves a major endogenous protective role. We now demonstrate that Parkin is highly expressed in both healthy platelets and diabetic platelets, compared to other mitochondria-enriched tissues such as the heart, muscle, brain, and liver. Abundance of Parkin in a small, short-lived anucleate cell suggest significance in various key processes. Through proteomics we identified 127 Parkin-interacting proteins in DM platelets and compared them to healthy controls. We assessed the 11 highest covered proteins by individual IPs and confirmed seven proteins that interacted with Parkin; VCP/p97, LAMP1, HADHA, FREMT3, PDIA, ILK, and 14-3-3. Upon further STRING analysis using GO and KEGG, interactions were divided into two broad groups: targeting platelet activation through (1) actions on mitochondria and (2) actions on integrin signaling. Parkin plays an important role in mitochondrial protection through mitophagy (VCP/p97), recruiting phagophores, and targeting lysosomes (with LAMP1). Mitochondrial β-oxidation may also be regulated by the Parkin/HADHA interaction. Parkin may regulate platelet aggregation and activation through integrin signaling through interactions with proteins like FREMT3, PDIA, ILK, and 14-3-3. Thus, platelet Parkin may regulate the protection (mitophagy) and stress response (platelet activation) in DM platelets. This study identified new potential therapeutic targets for platelet mitochondrial dysfunction and hyperactivation in diabetes mellitus.

## 1. Introduction

Diabetes mellitus (DM) is a progressive and chronic metabolic disorder characterized by hyperglycemia arising from impaired insulin levels, insulin sensitivity, and/or insulin activity. Currently, over 19.7 million adults in the USA have diagnosed DM, and an estimated 8.2 million have undiagnosed DM [1]. Cardiovascular disease is the major cause of morbidity and mortality among DM patients with approximately 65% of deaths caused by thrombotic events like myocardial and cerebrovascular ischemia and infarction [2]. Platelets play key roles in thrombotic occlusions of major vessels and tissue death.

Platelets are short-lived (7 to 10 days) circulating cells (2 to 4 μm) that contain many critical factors required for the regulation of thrombus formation, vascular homeostasis, and immune responses [3,4,5,6]. Platelets are capable of many fundamental cellular functions despite having no transcriptional capabilities (anucleate) including de novo protein synthesis [4,7,8] and programmed cell death [9,10]. Basal autophagy [11,12], which is distinct from induced autophagy [9,13,14], has only recently been described in platelets. This well-orchestrated process requires considerable energy for prepacking all the relevant mRNAs (no transcription) to maintain normal cellular function (basal autophagy) and for protection (induced autophagy) against severe oxidative stressors as observed with DM [13]. DM platelets have a highly induced protective autophagy processes including Parkin-dependent mitophagy [9,14].

Parkin is a Parkinson’s disease (PD)-related protein with several mutations identified in PD patients [15,16]. Parkin has ubiquitin E3 ligase activity and increased substrate ubiquitination via lysine 27, 29, 48, and 63 of ubiquitin [17,18,19]. Recent reports suggest that Parkin is inactivated by post-translational modifications like oxidation [14] and nitrosylation [16,20]. Additionally, Parkin regulates mitochondrial quality control through a well-orchestrated mitophagy process [21]. During mitochondrial damage, PTEN-induced kinase1 (PINK1) accumulates on damaged mitochondria and recruits Parkin, after which damaged mitochondria is then recognized by LC3-conjugated phagophore structures [21,22]. Parkin expression was increased in DM platelets and readily interacted with MsrB2 to remove damaged mitochondria [9,14].

Parkin is highly expressed in platelets compared to other mitochondrial-enriched tissues. However, the roles of Parkin remain largely unexplored in platelets. To determine alternative functions of Parkin in platelets, interacting proteins in healthy control and DM platelets were documented through literature investigations, we suggest functional associations between platelet aggregation, mitochondrial damage, mitophagy, and Parkin in DM platelets.

## 2. Results

We recently demonstrated that Parkin plays a key protective role against oxidative stress in platelets by inducing mitophagy [9,14]. The absence of Parkin during such stressors like DM and H_2_O_2_ leads to platelet apoptosis [9,14].

### 2.1. Parkin Interacts with Various Key Platelet Proteins

**Figure 1:**
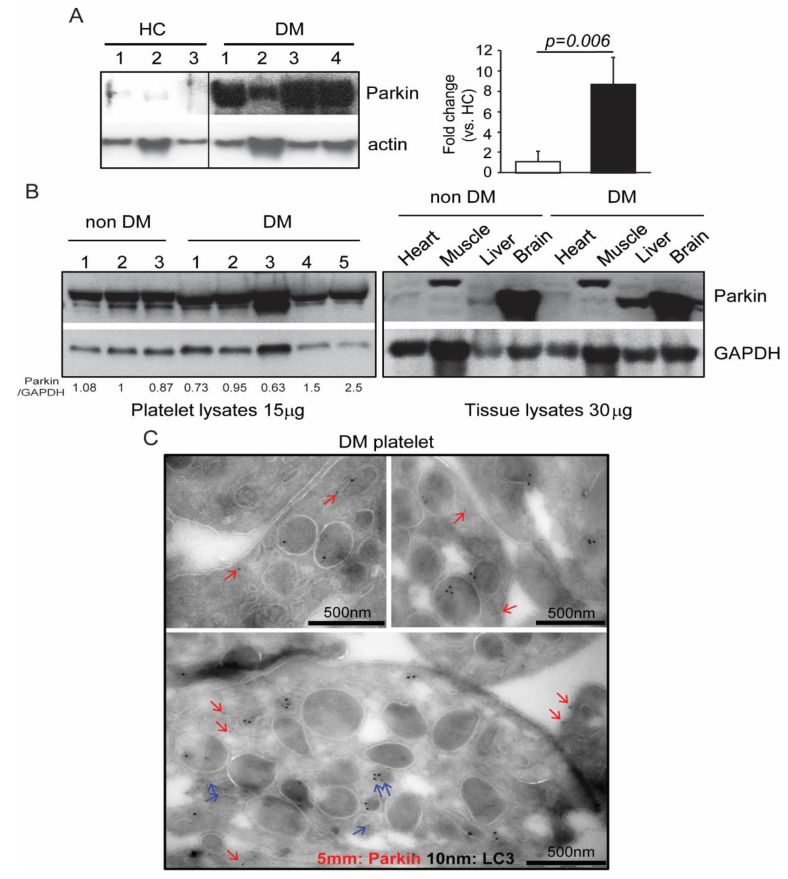
Parkin was highly expressed in platelets. (**A**) Western blot analysis of Parkin expression in human platelets (platelets isolated from three healthy control and four diabetic patients) (left). Quantification of Parkin in HC and DM (right) (**B**) Platelets, heart, muscle, liver, and brain tissues isolated from non-DM and DM mice (platelets isolated from three non-DM and five DM mice). Western blot analysis of Parkin in each sample with lanes representing individual mice. (**C**) Parkin and LC3 immuno-EM analysis of DM platelets where 5 nm dots indicate immunogold-labeled Parkin clusters, and 10 nm dots indicate immunogold-labeled LC3 clusters. Representative areas of clusters of gold labeling in DM patients (**A**–**C**) are presented. Blue arrows indicate mitochondrial and granular Parkin. Red arrows indicate parkin in other regions.

We previously verified that mitophagy induction in DM platelets occurs through a Parkin-dependent mechanism [9]. Surprisingly, Parkin is highly expressed in human DM platelet (Figure 1A) [9,14]. Although Parkin expression was only slightly increased in murine DM platelets, its expression was more than double that of other mitochondria-rich tissues like the heart, muscle, liver, and brain. (Figure 1B) [14]. Electron microscopy demonstrated that Parkin is localized within mitochondria and granules (blue arrows), cytosol, and cell membrane (red arrows) in DM platelets, further elaborating on its potentially diverse functions (Figure 1C). Mitochondrial Parkin also colocalizes with LC3, an autophagy marker (Figure 1C). No other functions beyond mitophagy induction in platelets have been reported for Parkin [14].

**Figure 2:**
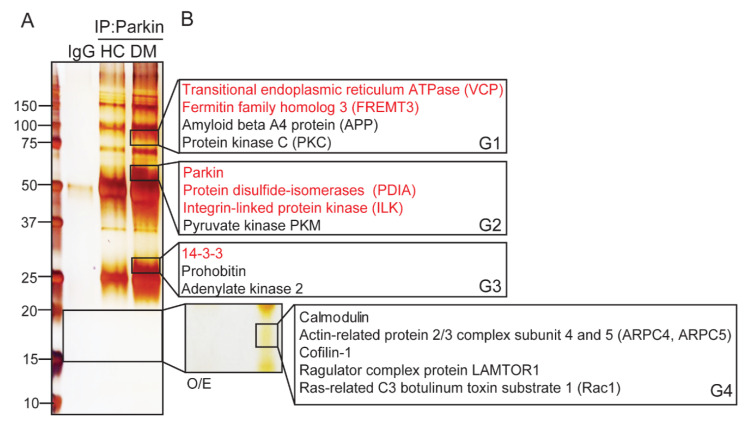
Identification of Parkin-interacting proteins in human DM platelets. (**A**) Immunoprecipitation of Parkin-interacting proteins in human DM platelets visualized by silver staining. We isolated 10 enriched bands from DM and compared it with HC for LC-MS/MS analysis. (**B**) Potential interacting proteins were analyzed by LC-MS/MS and using Mascot analysis.

To understand the various functions of Parkin in platelets, we identified Parkin-interacting proteins through immunoprecipitation (IP) with Parkin-specific antibodies and LC-MS/MS (Figure 2). We identified 127 interacting proteins (Table 1, Table 2 and Table 3). Coverage was more than 40% for 33 interacting proteins (Table 1). These proteins include contain platelet aggregating factors such as fibrinogen, coagulation factors, and platelet factors 4. Ubiquitin E2 ligase (UBE2V1), actin related (ARPC5 and cofilin-1), and mitochondria-related proteins (prohibitin, NDUFA2 and ATP5l) were also identified. Moreover, several isoforms of 14-3-3 isoforms were also identified. The 94 proteins with coverage below 40% are listed in Table 2 and Table 3.

**Figure 3:**
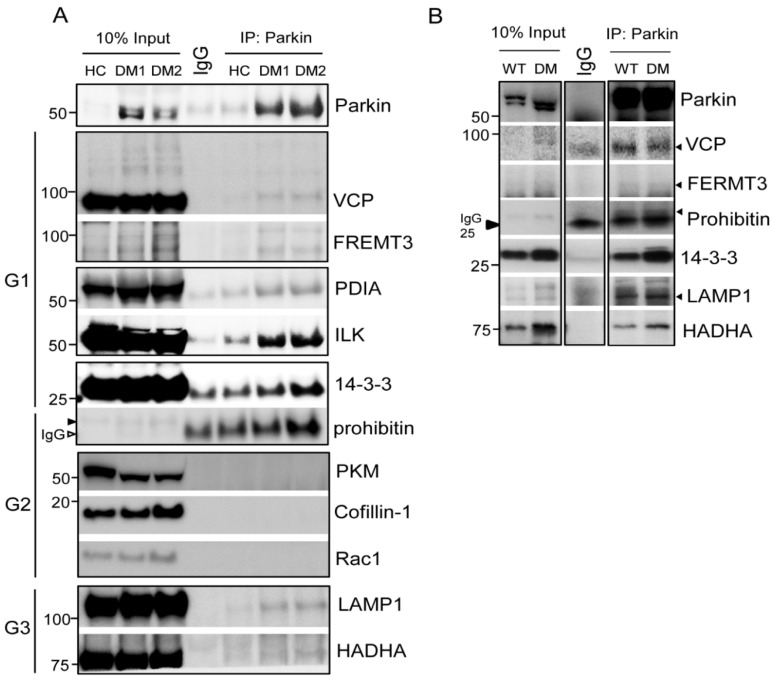
Confirmation of interactions between Parkin and selected target proteins in human and murine platelets. (**A**) Immunoprecipitation of each specific antibody in pooled human DM platelets (4 HC, 5DM1, and 6DM2). We incubated 500 μg protein lysates incubated with specific antibodies overnight at 4 °C with 10% input as control. G1 represents interacting protein groups from LC-MS/MS results. G2 represents the non-interacting proteins group in LC-MS/MS results. G3 represents protein that interacted with Parkin and that were not found in the LC-MS/MS results. (**B**) Immunoprecipitation of each specific antibody in pooled murine platelets (3 WT and 5DM). We incubated 500 μg protein lysates incubated with specific antibodies overnight at 4 °C with 10% input as control.

We confirmed interactions between Parkin and the identified proteins in human and murine platelets (Figure 3). Initially, we selected 11 candidates for Parkin interaction verification. VCP/p97, FREMT3, PDIA, ILK, and 14-3-3 (G1) interacted with Parkin in human platelets but prohibitin, PKM, cofillin-1, and Rac1 did not (G2) (Figure 3A). Additionally, the mitochondrial β-oxidation related protein Hydroxyacyl-CoA dehydrogenase/3-ketoacyl-CoA thiolase/enoyl-CoA hydratase alpha subunits (HADHA), and LAMP1 (mitophagy related) (G3) also interacted with Parkin in platelets (Figure 3A). VCP/p97, FERMT3, LAMP1, HADHA, 14-3-3, and prohibitin interacted with Parkin in murine platelets (Figure 3B).

### 2.2. Determining the Potential Roles of Platelet Parkin-Interacting Proteins through Literature Analysis

#### 2.2.1. Parkin Plays an Important Role in Mitochondrial Protection through Mitophagy

##### Transitional Endoplasmic Reticulum ATPase (VCP/p97)

VCP/p97 is a hexameric protein of the AAA (ATPases associated with diverse cellular activities) family which generally utilizes energy from ATP hydrolysis [23]. VCP/p97 has been linked to various membrane trafficking processes, including Golgi reassembly post-mitosis [24] and control of lipid droplet biogenesis [25]. Emerging evidence has connected VCP/p97 to lysosomal protein degradation through its ability to facilitate cargo sorting via the endosomal pathway and autophagy [26,27,28]. One VCP/p97 mutation causes a rare multisystem disease, IBMPFD (inclusion body myopathy with Paget’s disease and frontotemporal dementia) [28]. Several recent studies reported the involvement of VCP/p79 in mitophagy [29,30]. Here, we identified that VCP/p97 interacts with Parkin in DM platelets, possibly regulating the mitophagy process (VCP/p97: 78.5% coverage, Table 1).

##### Lysosomal-Associated Membrane Protein 1 (LAMP1)

LAMP1 is a well-known lysosomal protein that we previously confirmed to colocalize with Parkin and LC3 in DM platelets [9,14] and, again, in this study through IP (Figure 3). Although not identified in the LC-MS/MS, LAMP1 was used to confirm Parkin’s role in autophagy activation (Table 1, Table 2 and Table 3). This highlights the potential deficiencies and false negatives of the LC-MS/MS, potentially due to the complex processing.

##### Mitochondrial Three Functional Protein A (TFPα, HADHA)

TFPα is a multienzyme mitochondrial complex harboring three major enzymes from the β-oxidation cycle of long-chain fatty acids. TFPα deficiencies present in neonates as a severe cardiac phenotype, often with death in the first weeks. Deficiency is related to maternal HELLP (hydrolysis, elevated liver enzyme and low platelets) syndrome and reduced birth weight [31]. HADHA is involved in long-chain fatty acid-induced autophagy of intestinal epithelial cells and is therefore proposed as a new therapeutic target for inflammatory bowel disease (IBD) [32]. A functional relationship may exist between Parkin and HADHA.

##### Prohibitin (PHB, Murine Only)

Two members of the prohibitin family, PHB1 and PHB2, are highly homologous proteins localized to the mitochondrial inner membrane [33,34]. The PHB complexes perform diverse functions in mitochondria, including regulation of membrane protein degradation, chaperones, regulation of oxidative phosphorylation, maintenance of mitochondrial genetic stability, and regulation of mitochondrial morphology [33,34]. PHB1 and 2 also function as autophagy receptors [35,36,37,38]. PHB2 directly interacts with LC3 potentially regulating the mitophagy process [37]. In platelets, prohibitin is expressed in membranes and is involved in PAR1-mediated platelet aggregation [39]. Here, we confirmed that prohibitin can interact with Parkin in murine platelets but not in human DM platelets (Figure 3B).

#### 2.2.2. Parkin Regulates DM Platelets through Protein Interactions with Integrin Complex Proteins

**Figure 4:**
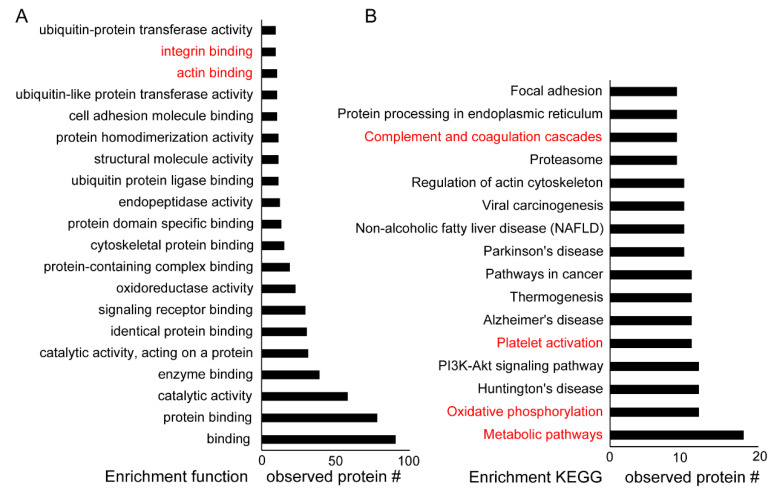
Enriched function (GO) and KEGG analysis of identified proteins. STRING (functional protein association networks) used in the grouping of identified proteins. (**A**) Top 20 enriched functions of Parkin-interacting proteins in human DM platelets. (**B**) Top 16 enriched KEGG pathways of Parkin-interacting proteins in human DM platelets.

We analyzed the enriched function (GO) and KEGG pathways using the identified proteins (Figure 4). Parkin-interacting proteins were associated with integrin binding, signaling, and ubiquitin proteasome system in DM platelets (Figure 4A). Parkin may participate in the metabolic pathway and oxidative phosphorylation of DM platelets as well as platelet activation and the coagulation cascade (Figure 4B). Parkin is localized to the mitochondrial outer membrane during mitophagy activation. Furthermore, Parkin may be associated with integrin signaling through 14-3-3, Amyloid beta A4 protein [1], Calreticulin (CALR), EMILIN1, fermitin family homolog3 (FERMT3), integrin-linked protein kinase [1], integrin beta 1 and 3 (ITGB1 and ITGB3), protein disulfide isomerase (PDI/P4HB), thrombospondin-1 (THBS1), and von Willebrand factor (VWF) in platelets (Table 1, Table 2 and Table 3). Our study confirmed the interaction between Parkin and 14-3-3, FREMT3, PDI, and ILK (Figure 3). Parkin may integrate outside-in signaling in platelets and may induce platelet activation through these interactions.

##### 14-3-3

**Figure 5:**
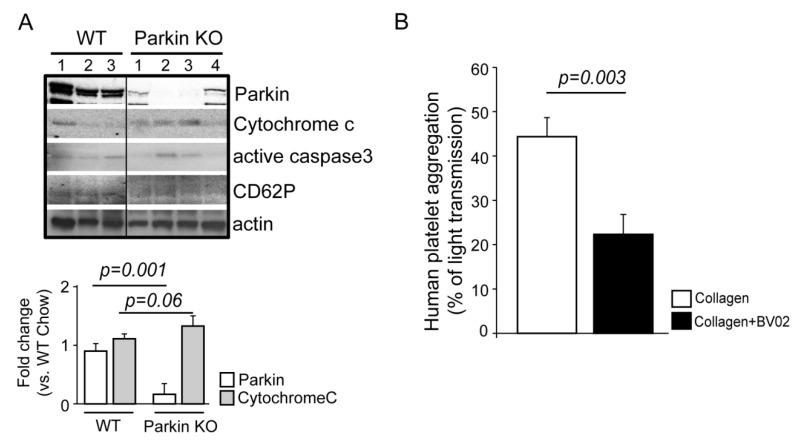
Parkin associated with apoptosis and platelet aggregation. (**A**) Western blot analysis of Parkin KO mice platelets. Parkin, Cytochrome C, active caspase3, and CD62P antibodies were used for this experiment. The numbers indicate individual mice. (**B**) Quantification of each group using ImageJ software. (**B**) Collagen induced platelet aggregation in human platelets (*n* = 3) after BVO2 (14-3-3 inhibitor) treatment.

Hyperactivation and hyperaggregation are well-known phenotypes of DM platelets [5,6]. Additionally, 14-3-3 is involved in platelet aggregation with GPIB-IX-V [40,41]. The 14-3-3 proteins family are highly conserved intracellular proteins with several isoforms: β, ε, ζ, γ, η, τ, and σ. These isoforms associate as homo- and heterodimers interact with over 200 different proteins including serine and threonine phosphorylated intracellular proteins. The 14-3-3 proteins interact with diverse intracellular molecules, signaling proteins, metabolic enzymes, cytoskeletal proteins, transcription factors, and apoptosis-related proteins and can regulate platelet mitochondrial respiratory [42,43,44]. It has also been associated with neuronal diseases [36,45,46]; 14-3-3 η is a well-known negative regulator of Parkin E3 activity through direct interactions in mice brains [46]. Six 14-3-3 isoforms have been detected in human platelets including β, ε, ζ, γ, η and τ, with ζ and γ expressed at high levels [43,47]. The first identified 14-3-3 ζ binds to the cytoplasmic tails of GPIbα and GPIbβ [40,48] while other reports have suggested that the GPIb-14-3-3ζ interactions can promote VWF-dependent integrin α_IIβ_β_3_-activation and cell spreading [48,49]. Mice that are 14-3-3ζ-deficient exhibit defective pro-coagulant function reduced arterial thrombosis, reduced thrombin generation, and pulmonary embolism in vivo. Platelet bioenergetics has revealed enhanced mitochondrial respiratory reserve capacities in 14-3-3ζ-deficient platelets that correlated with sustained levels of metabolic ATP levels. Moreover, 14-3-3ζ serves as an important regulator of platelet bioenergetic functioning and, therefore, also pro-coagulant and thrombosis in vivo [47]. The LC-MS/MS results indicated that Parkin interacted with 14-3-3 ε, λ, θ, η, δ, ζ, α, and β in DM platelets (Table 1). Therefore, Parkin may interact indirectly with GPIbα-V-IX, integrin beta3, and cellular signaling through interactions with diverse 14-3-3 isoforms. The 14-3-3 inhibitor, BVO2, inhibited the GPIb-IX-V complex and collagen induced platelet aggregation (Figure 5B). Parkin may regulate integrin-14-3-3-induced platelet activation. Collectively, our results and previous reports, predict that Parkin may regulate platelet aggregation through 14-3-3 and GPIb-IX-V complexes. Furthermore, Parkin participates in platelet activation through integrin signaling.

##### Fermitin Family Homolog 3 (FERMT3: URP2/Kindlin-3)

The kindlin family members, including kindlin-1, kindlin-2, and kindlin-3, have high sequence homology but display different tissue expression patterns [50]. These proteins strongly associate with human diseases, as a lack of kindlin-1 in humans cause Kindler syndrome. Kindlin-2 deficiencies have not been reported with lethal consequences [51,52,53]. Kindlin-3 deficiencies display severe bleeding and recurrent infections due to the dysfunctional integrin in platelet and leukocytes [54,55,56]. Kindlin-3 deficiencies are key to supporting integrin activation and platelet thrombus formation [50]. Parkin interacts with kindlin-3 in human and murine DM platelets (kindlin-3/FERMT3: 55.8% coverage, Figure 3). Based on this and Parkin’s role in mitophagy, we suggest that FERMT3 is involved in platelet mitophagy and thrombus formation through integrin signaling with Parkin.

##### Protein Disulfide-Isomerase (PDI, P4HB)

Protein disulfide isomerase (PDI) was identified 20 years ago as an endoplasmic reticulum protein that facilitates the formation of correct disulfide bonds in nascent proteins [57]. There are more than 20 members of the PDI, seven containing a CGHC-active site [57]. Among CGHC-active site members, four are associated with platelet function and thrombosis (PDI, ERp57, ERp72, and ERp5) [57]. PDI was the first from this protein family identified in integrin-mediated platelet aggregation, adhesion, and thrombosis [58,59]. Here, in our LC-MS/MS results, we identified PDIA 1, 3, 5, and 6 as Parkin-interacting proteins (Table 1, Table 2 and Table 3). Parkin-associated integrin-mediated platelet function, homeostasis, and thrombosis are dependent on PDIs to form appropriate disulfide bonds.

##### Integrin Linked Kinase

Integrin-linked kinase was reported to interact with the cytoplasmic tail of β-integrin subunits and its serine/threonine kinase activity is upregulated through platelet stimulation [60,61,62]. ILKs have functions as adaptor proteins, interacting and regulating β1 and β3 integrin subunits [63]. ILK-deficient mice exhibit reduced platelet activation and aggregation and increased bleeding [60,62]. ILK regulates the rate of platelet activation rates and is essential for the formation of stable thrombi by controlling platelet response rates to collagen via GPVI [62]. From this, Parkin may regulate platelet functioning by binding with integrin and its related proteins like ILK (49.1% coverage, Table 1).

### 2.3. New Functions of Parkin and Parkin-Interacting Proteins in Platelets

Through GO and KEGG analysis in conjunction with literature reviews, we present and support additional Parkin functions through confirmed Parkin-interacting proteins. Key platelet functions including activation, aggregation, and mitochondrial functions appear to be regulated, at least partially, by Parkin. We previously verified Parkin-dependent mitophagy activation in a T2DM mouse model [9,14]. To confirm the importance of Parkin in platelets, we incorporated Parkin KO mice platelets. In Parkin KO mice platelets, there was increased cytochrome C and active caspase3 indicative of increased platelet apoptosis (Figure 5A). The platelet activation maker, CD62P (pSelectin), was slightly decreased but not significantly. The LC-MS/MS analysis verified the functional relationship between platelet activation-associated proteins and Parkin (Table 1, Table 2 and Table 3), suggesting that Parkin regulates platelet activation. We then used a 14-3-3 inhibitor to interrupt the interaction with Parkin (Figure 5B) which decreased platelet aggregation induced by collagen through 14-3-3 inhibitor treatment.

## 3. Discussion

**Figure 6:**
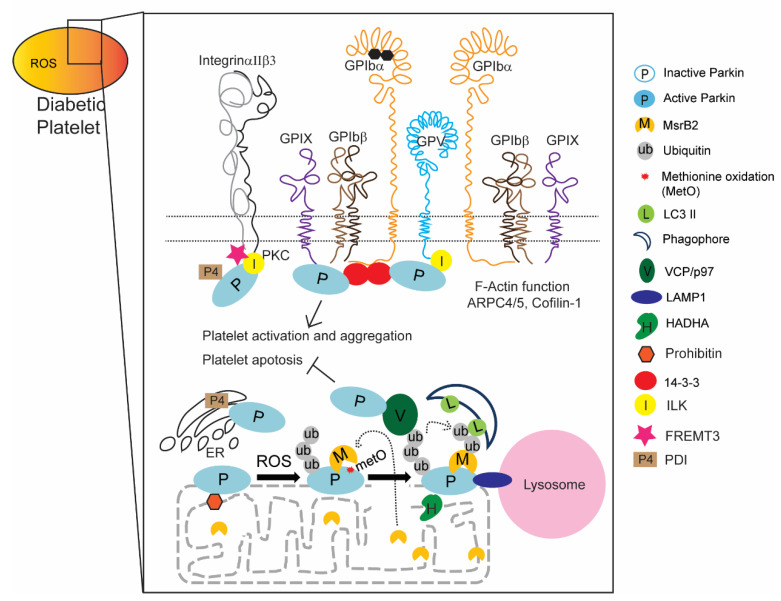
Summary of Parkin functions in DM platelets.

We previously verified that Parkin expression levels were high in HC platelets, even more so in DM [9,14] and underwent post-translational modification (MetO) under oxidative stress [14]. Cysteine oxidation is significantly increased in PD and includes methionine oxidation [64]. We confirmed that the ubiquitylation and methionine oxidation that occurs in Parkin lead to mitophagy. In this study, we aimed to uncover and verify other key platelet functions regulated by Parkin through the identification of Parkin-interacting proteins using IP and LC-MS/MS. Through this proteomic approach, we provided experimental and literature evidence that Parkin may regulate various processes beyond mitophagy, including integrin-dependent signaling, mitochondrial energy metabolism, platelet activation/aggregation, and ER-mitochondrial cross-talk (Figure 6). Furthermore, surface membrane receptor interactions with Parkin provide a possible link between external signaling and internal cellular processes.

Parkin transfers the changes in the external environment, like diabetes, to the internal environment by interacting with membrane receptor proteins, like integrin, inducing mitophagy. Parkin also indirectly interacts with cytosolic proteins, F-actin functional ARP4/5, and directly with VCP/p97 associated with mitophagy activation. PINK1 is well-known to interact with Parkin and VCP/p97, after which this complex regulates dendritic arborization [65]. Based on these results, we hypothesize that Parkin regulates organelle cross-talk potentially transferring external signals to internal platelet environments and cell–cell cross-talk among other cells. Proteomic analyses also suggest that Parkin participates in diverse processes and mitophagy induction in DM platelets, like phagophore recruitment to damaged mitochondria through interactions with MsrB2 [14] and regulation of autolysosome formation through interactions with LAMP1. Parkin-deficient mice exhibit increased apoptotic platelets compared with healthy control mice (Figure 5A) [9]. Parkin interacts with 14-3-3, PDIA, FREMT3, ILK, and F-actin-related proteins (well-known proteins associated with platelet activation and aggregation), and so we suggest that Parkin is also involved with platelet aggregation and activation (Figure 6). In DM platelets, mitochondrial outer membrane is disrupted with mitochondrial protein release and exposed inner membrane proteins [14]. Consequently, the interaction between Parkin and HADHA and prohibitin may occur in human and murine DM platelets (Table 2), suggesting that Parkin is likely to participate in platelet energy metabolism in DM (Figure 6).

In conclusion, our results suggest that Parkin with its many actions on platelet mitochondria, activation, and aggregation may be a therapeutic target for antiplatelet treatment. However, further detailed molecular studies are required that focus on the individual binding partners of Parkin in DM platelets.

## 4. Material and Methods

### 4.1. Preparation of Human Platelet

Venous blood was drawn from healthy and patients at Yale University School of Medicine (HIC#1005006865) from multiple outpatient clinics including the cardiovascular, diabetes, and neurology clinics. Informed consent was obtained from all subjects, and the experiments conformed to the principles set out in the WMA Declaration of Helsinki and the Department of Health and Human Services Belmont Report (IRB#1006006865, 5/11/2017). All healthy subjects were free from medication or diseases known to interfere with platelet function [5,6,9]. Upon informed consent, a venous blood sample (approximately 20 cc) was drawn by standard venipuncture and collected into tubes containing 3.8% trisodium citrate (*w*/*v*). Blood samples were prepared as previously described [66]. Platelet-rich plasma (PRP) was obtained by differential centrifugation. Purity of platelet preparation was determined by Western blot analysis using platelet markers (CD41), monocyte markers (CD14), and red blood cell markers (CD235a) [9].

### 4.2. Preparation of Mice Platelets

Blood (0.7–1 mL) was directly aspirated from the right cardiac ventricle into 1.8% sodium citrate (pH 7.4) in WT (C57Bl/6) and diabetic mice (mice were 8 weeks of age; STZ injected for 5 days followed by high-fat diet for 12 weeks). Citrated blood from several mice with identical genotype was pooled and diluted with equal volume of HEPES/Tyrode’s buffer. PRP was prepared by centrifugation at 100 g for 10 min and then used for Immunoprecipitation and Western blotting. All mice were of C57Bl/6 background (WT and Parkin whole-body knockout). The experiment of Parkin KO mice experiment was performed at the Yale Animal Facility and 300 George St. New Haven, CT, USA, under the supervision of YARC (Yale Animal Resources Center) and Rita Weber (Animal facility manager, YARC). All experiments were performed in accordance with guidelines and regulations as outlined by IACUC (the Yale Institutional Animal Care and Use Committee) under the approved protocol (IACUC# 2017-11413 (3/31/2017)). Diabetic mice were generated in a Korea CDC animal facility. All experiments were performed in accordance with guidelines and regulations under the approved protocol (#KCDC-109-18-2A (2019), #KCDC-116-19-2A (2020), and #KCDC-115-19-2A (2020)).

### 4.3. Western Blotting

Standard Western blot analysis protocols were used. A 10% Input of IP lysates was loaded in each well as loading control. We used specific individual antibodies and dilutions (Parkin: abcam #ab15954 (1:1000), VCP: Invitrogen #MA3-004 (1:1000), FREMT3: abcam #ab68040 (1:1000), PDIA: abcam #ab2792 (1:1000), ILK: abcam #ab52480 (1:1000), 14-3-3: Thermo Fisher scientific #51-0700 (1:1000), prohibitin: abcam #ab28172 (1:1000), LAMP1: cell signaling #9091 (1:1000), HADHA: abcam #ab203114 (1:1000), PKM: abcam #ab137791 (1:1000), cofillin-1: GeneTex #GTX102156 (1:1000), Rac1: Sigma–Aldrich #SAB4502560 (1:1000), GAPDH: cell signaling #3683 (1:1000), Cytochrome C: abcam #ab90529 (1:2000), CD62P: abcam #182135 (1:1000), Actin: Santa Cruz #SC47778 (1:1000)).

### 4.4. Immunoprecipitation

The 500 μg pulled healthy/DM platelet lysates and cell lysates (after transient transfection) were mixed with 1 μg specific target antibodies and the same species IgG control with HC) and incubated overnight at 4 °C. Then 50% slurry protein A sepharose beads and 50% slurry protein G sepharose beads were mixed 1:1. Next, 30 μL of the 50% slurry washed A/G beads with lysates/antibodies mixture was incubated for 1 h at 4 °C. After 3 more washes with lysis buffer, we used 1–10% lysates were used for the input. We pooled 4 healthy subjects and 11 human DM platelets (5 pooled in DM1 and 6 pooled in DM2) for Parkin immunoprecipitation (Figure 3A) and pooled 3 WT and 5 DM mice platelets (Figure 3B).

### 4.5. Silver Staining and LC-Mass Analysis

Mass spectrometry was performed according to the manufacturer’s protocols by Pierce silver stain, and individual bands were excised for LC-MS/MS after silver staining. In-gel digestion, LC-MS/MS, and peptide identification were performed by Yale MS & Proteomics Resource.

In-Gel Digestion: Silver-stained gel bands were treated with 5% acetic acid for 10 min with rocking. The acid was removed, and the bands were covered with freshly prepared destaining solution (made fresh by mixing in a 1:1 ratio stock solutions of 30 mM potassium ferricyanide in water and 100 mM sodium thiosulfate in water) until the brownish color disappeared. The bands were then rinsed three times with 0.5 mL of water for 5 min to remove the acid and chemical reducing agents. The gel bands were cut into small pieces and washed for 30 min on a tilt-table with 450 µL 50% acetonitrile/100 mM NH_4_HCO_3_ (ammonium bicarbonate) followed by a 30 min wash with 50% acetonitrile/12.5 mM NH_4_HCO_3_. The gel bands were shrunk by the brief addition then removal of acetonitrile, and then dried by speed vacuum. Each sample was resuspended in 100 µL of 25 mM NH_4_HCO_3_ containing 0.5 µg of digestion grade trypsin (Promega, V5111) and incubated at 37 °C for 16 h. Supernatants containing tryptic peptides were transferred to new Eppendorf tubes and the gel bands were extracted with 300 µL of 80% acetonitrile/0.1% trifluoroacetic acid for 15 min. Supernatants were combined and dried by speed vacuum. Peptides were dissolved in 24 µL MS loading buffer (2% acetonitrile, 0.2% trifluoroacetic acid) with 5 µL injected for LC-MS/MS analysis.

LC-MS/MS on the Thermo Scientific Q Exactive Plus: LC-MS/MS analysis was performed on a Thermo Scientific Q Exactive Plus equipped with a Waters nanoAcquity UPLC system utilizing a binary solvent system (A: 100% water, 0.1% formic acid; B: 100% acetonitrile, 0.1% formic acid). Trapping was performed at 5 µL/min, 97% Buffer A for 3 min using a Waters Symmetry^®^ C18 180 µm and 20 mm trap column (Waters, USA). Peptides were separated using an ACQUITY UPLC PST [67] C18 nanoACQUITY Column 1.7 µm, 75 µm × 250 mm (37 °C) and eluted at 300 nL/min with the following gradient: 3% buffer B at initial conditions; 5% B at 1 min; 35% B at 50 min; 50% B at 60 min; 90% B at 65 min; 90% B at 70 min; return to initial conditions at 71 min. MS was acquired in profile mode over the 300–1700 m/z range using 1 microscan, 70,000 resolution, AGC target of 3E6, and a maximum injection time of 45 ms. Data dependent MS/MS were acquired in centroid mode on the top 20 precursors per MS scan using 1 microscan, 17,500 resolution, AGC target of 1E5, maximum injection time of 100 ms, and an isolation window of 1.7 m/z. Precursors were fragmented by HCD activation with collision energy of 28%. MS/MS were collected on species with an intensity threshold of 2E4, charge states 2–6, and peptide match preferred. Dynamic exclusion was set to 20 s.

Peptide Identification: Tandem mass spectra were extracted by Proteome Discoverer software (version 1.3, Thermo Scientific) and searched in-house using the Mascot algorithm (version 2.6.0, Matrix Science). The data were searched against the SwissProt database (version 2017_01) with taxonomy restricted to Homo sapiens (20,172 sequences). Search parameters included trypsin digestion up to 2 missed cleavages, peptide mass tolerance of 10 ppm, MS/MS fragment tolerance of 0.02 Da, and methionine oxidation and propionamide adduct to cysteine as variable modifications. Normal and decoy database searches were run with the confidence level set to 95% (*p* < 0.05).

### 4.6. Platelet Aggregation Test

Platelet suspensions were incubated with BV02 (2-(2,3-Dihydro-1,5-dimethyl-3-oxo-2-phenyl-1H-pyrazol-4-yl)-2,3-dihydro-1,3-dioxo-1H-isoindole-5-carboxylic acid, 14-3-3 inhibitor, 10 nM) for 1 h. Platelet aggregation was monitored at 37 °C with constant stirring (1200 rpm) in a dual-channel lumi-aggregometer (model 700; Chrono-Log). Platelet aggregation was measured as the increase in light transmission for 10 min, starting with the addition of 2 μL of 1 mg/mL collagen (Chrono-Log) to a 500 μL reaction as a pro-aggregatory stimulus; the final concentration was 4 μg/mL. The maximum aggregation was expressed as a percentage of maximum light transmission with non-stimulated PRP of 0% and PPP of 100%.

## Figures and Tables

**Table 1:** Identification of Parkin-interacting proteins in DM platelets (above 40% coverage).

Protein ID	Protein Name	MW (Da)	% Coverage
FIBG_HUMAN	Fibrinogen gamma chain OS = Homo sapiens GN = FGG PE = 1 SV = 3	51,479	78.8
TERA_HUMAN	Transitional endoplasmic reticulum ATPase OS = Homo sapiens GN = VCP PE = 1 SV = 4	89,266	78.5
FIBB_HUMAN	Fibrinogen beta chain OS = Homo sapiens GN = FGB PE = 1 SV = 2	55,892	75.4
1433Z_HUMAN	14-3-3 protein zeta/delta OS = Homo sapiens GN = YWHAZ PE = 1 SV = 1	27,728	74.7
ARPC5_HUMAN	Actin-Related protein 2/3 complex subunit 5 OS = Homo sapiens GN = ARPC5 PE = 1 SV = 3	16,310	72.2
1433F_HUMAN	14-3-3 protein eta OS = Homo sapiens GN=YWHAH PE = 1 SV = 4	28,201	69.1
DYL1_HUMAN	Dynein light chain 1, cytoplasmic OS = Homo sapiens GN = DYNLL1 PE = 1 SV = 1	10,359	64
1433B_HUMAN	14-3-3 protein beta/alpha OS = Homo sapiens GN = YWHAB PE = 1 SV = 3	28,065	63
GSTO1_HUMAN	Glutathione S-transferase omega-1 OS=Homo sapiens GN = GSTO1 PE = 1 SV = 2	27,548	61.4
TPM4_HUMAN	Tropomyosin alpha-4 chain OS=Homo sapiens GN = TPM4 PE = 1 SV = 3	28,504	60.1
1433E_HUMAN	14-3-3 protein epsilon OS = Homo sapiens GN = YWHAE PE = 1 SV = 1	29,155	59.2
TSP1_HUMAN	Thrombospondin-1 OS = Homo sapiens GN = THBS1 PE = 1 SV = 2	129,300	58.1
FIBA_HUMAN	Fibrinogen alpha chain OS = Homo sapiens GN = FGA PE = 1 SV = 2	94,914	56.9
URP2_HUMAN	Fermitin family homolog 3 OS = Homo sapiens GN = FERMT3 PE = 1 SV = 1	75,905	55.8
PRDX6_HUMAN	Peroxiredoxin-6 OS = Homo sapiens GN = PRDX6 PE = 1 SV = 3	25,019	54
CAP1_HUMAN	Adenylyl cyclase-associated protein 1 OS=Homo sapiens GN = CAP1 PE = 1 SV = 5	51,869	53.5
S10A8_HUMAN	Protein S100-A8 OS = Homo sapiens GN = S100A8 PE = 1 SV = 1	10,828	52.7
1433T_HUMAN	14-3-3 protein theta OS = Homo sapiens GN = YWHAQ PE = 1 SV = 1	27,747	51.4
F13A_HUMAN	Coagulation factor XIII A chain OS = Homo sapiens GN = F13A1 PE = 1 SV = 4	83,215	51.2
PDIA5_HUMAN	Protein disulfide-isomerase A5 OS=Homo sapiens GN = PDIA5 PE = 1 SV = 1	59,556	50.5
PHB_HUMAN	Prohibitin OS = Homo sapiens GN = PHB PE = 1 SV = 1	29,786	50.4
ILK_HUMAN	Integrin-linked protein kinase OS = Homo sapiens GN =I LK PE = 1 SV = 2	51,386	49.1
MMRN1_HUMAN	Multimerin-1 OS = Homo sapiens GN = MMRN1 PE = 1 SV = 3	138,023	48.4
COF1_HUMAN	Cofilin-1 OS = Homo sapiens GN = CFL1 PE = 1 SV = 3	18,491	47
1433G_HUMAN	14-3-3 protein gamma OS = Homo sapiens GN = YWHAG PE = 1 SV = 2	28,285	47
LTOR1_HUMAN	Ragulator complex protein LAMTOR1 OS = Homo sapiens GN = LAMTOR1 PE = 1 SV = 2	17,734	45.3
UB2V1_HUMAN	Ubiquitin-conjugating enzyme E2 variant 1 OS = Homo sapiens GN = UBE2V1 PE = 1 SV = 2	16,484	44.2
CALR_HUMAN	Calreticulin OS = Homo sapiens GN = CALR PE = 1 SV = 1	48,112	43.9
PLF4_HUMAN	Platelet factor 4 OS = Homo sapiens GN = PF4 PE = 1 SV = 2	10,838	43.6
PDIA6_HUMAN	Protein disulfide-isomerase A6 OS = Homo sapiens GN = PDIA6 PE = 1 SV = 1	48,091	42
ATP5I_HUMAN	ATP synthase subunit e, mitochondrial OS = Homo sapiens GN = ATP5I PE = 1 SV = 2	7928	40.6
EMIL1_HUMAN	EMILIN-1 OS = Homo sapiens GN = EMILIN1 PE = 1 SV = 2	106,601	40.4
NDUA2_HUMAN	NADH dehydrogenase [ubiquinone] 1 alpha subcomplex subunit 2 OS = Homo sapiens GN = NDUFA2 PE = 1 SV = 3	10,915	40.4

**Table 2:** Identification of Parkin-interacting proteins in DM platelets (39~20% coverage).

Protein ID	Protein Name	MW (Da)	% Coverage
PDE5A_HUMAN	cGMP-specific 3′,5′-cyclic phosphodiesterase OS = Homo sapiens GN = PDE5A PE = 1 SV = 2	99,921	38.7
CXCL7_HUMAN	Platelet basic protein OS = Homo sapiens GN = PPBP PE = 1 SV = 3	13,885	38.3
ATP5L_HUMAN	ATP synthase subunit g, mitochondrial OS = Homo sapiens GN = ATP5L PE = 1 SV = 3	11,421	37.9
S10A9_HUMAN	Protein S100-A9 OS = Homo sapiens GN = S100A9 PE = 1 SV = 1	13,234	37.7
NDUA4_HUMAN	Cytochrome c oxidase subunit NDUFA4 OS = Homo sapiens GN = NDUFA4 PE = 1 SV = 1	9,364	37
CALM_HUMAN	Calmodulin OS = Homo sapiens GN = CALM1 PE = 1 SV = 2	16,827	36.9
S10A4_HUMAN	Protein S100-A4 OS = Homo sapiens GN = S100A4 PE = 1 SV = 1	11,721	36.6
MIC60_HUMAN	MICOS complex subunit MIC60 OS = Homo sapiens GN = IMMT PE = 1 SV = 1	83,626	35.6
FINC_HUMAN	Fibronectin OS = Homo sapiens GN = FN1 PE = 1 SV = 4	262,460	34.5
NEXN_HUMAN	Nexilin OS = Homo sapiens GN = NEXN PE = 1 SV = 1	80,609	34.5
ITB3_HUMAN	Integrin beta-3 OS = Homo sapiens GN = ITGB3 PE = 1 SV = 2	87,000	33.6
PSA7_HUMAN	Proteasome subunit alpha type-7 OS = Homo sapiens GN = PSMA7 PE = 1 SV = 1	27,870	33.1
SAMP_HUMAN	Serum amyloid P-component OS = Homo sapiens GN = APCS PE = 1 SV = 2	25,371	32.7
SKAP2_HUMAN	Src kinase-associated phosphoprotein 2 OS = Homo sapiens GN = SKAP2 PE = 1 SV = 1	41,191	32.3
NUBP2_HUMAN	Cytosolic Fe-S cluster assembly factor NUBP2 OS = Homo sapiens GN = NUBP2 PE = 1 SV = 1	28,807	31.4
SSBP_HUMAN	Single-Stranded DNA-binding protein, mitochondrial OS = Homo sapiens GN = SSBP1 PE = 1 SV = 1	17,249	31.1
GPIX_HUMAN	Platelet glycoprotein IX OS = Homo sapiens GN = GP9 PE = 1 SV = 3	19,034	30.5
A4_HUMAN	Amyloid beta A4 protein OS = Homo sapiens GN = APP PE = 1 SV = 3	86,888	30
PSME2_HUMAN	Proteasome activator complex subunit 2 OS = Homo sapiens GN = PSME2 PE = 1 SV = 4	27,384	29.3
CXCL7_HUMAN	Platelet basic protein OS = Homo sapiens GN = PPBP PE = 1 SV = 3	13,885	27.3
KAD2_HUMAN	Adenylate kinase 2, mitochondrial OS=Homo sapiens GN = AK2 PE = 1 SV = 2	26,461	27.2
UB2L3_HUMAN	Ubiquitin-conjugating enzyme E2 L3 OS = Homo sapiens GN = UBE2L3 PE = 1 SV = 1	17,850	26.6
KPYM_HUMAN	Pyruvate kinase PKM OS = Homo sapiens GN = PKM PE = 1 SV = 4	57,900	26.2
DHB4_HUMAN	Peroxisomal multifunctional enzyme type 2 OS = Homo sapiens GN = HSD17B4 PE = 1 SV = 3	79,636	25.7
NDUA7_HUMAN	NADH dehydrogenase (ubiquinone) 1 alpha subcomplex subunit 7 OS =Homo sapiens GN = NDUFA7 PE =1 SV=3	12,544	25.7
PSA3_HUMAN	Proteasome subunit alpha type-3 OS = Homo sapiens GN = PSMA3 PE = 1 SV = 2	28,415	24.7
PRDX1_HUMAN	Peroxiredoxin-1 OS = Homo sapiens GN = PRDX1 PE = 1 SV = 1	22,096	23.6
PLMN_HUMAN	Plasminogen OS = Homo sapiens GN = PLG PE = 1 SV = 2	90,510	23.5
UBP5_HUMAN	Ubiquitin carboxyl-terminal hydrolase 5 OS = Homo sapiens GN = USP5 PE = 1 SV = 2	95,725	23.3
HECD3_HUMAN	E3 ubiquitin-protein ligase HECTD3 OS = Homo sapiens GN = HECTD3 PE = 1 SV = 1	97,051	22.8
CAN1_HUMAN	Calpain-1 catalytic subunit OS = Homo sapiens GN = CAPN1 PE = 1 SV = 1	81,838	22.5
RGS18_HUMAN	Regulator of G-protein signaling 18 OS = Homo sapiens GN = RGS18 PE = 1 SV = 1	27,565	22.1
PSME1_HUMAN	Proteasome activator complex subunit 1 OS = Homo sapiens GN = PSME1 PE = 1 SV = 1	28,705	22.1
PDIA1_HUMAN	Protein disulfide-isomerase OS = Homo sapiens GN = P4HB PE = 1 SV = 3	57,081	22
GPX1_HUMAN	Glutathione peroxidase 1 OS = Homo sapiens GN = GPX1 PE = 1 SV = 4	22,075	21.7
PDIA3_HUMAN	Protein disulfide-isomerase A3 OS = Homo sapiens GN = PDIA3 PE = 1 SV = 4	56,747	21
VWF_HUMAN	von Willebrand factor OS = Homo sapiens GN = VWF PE = 1 SV = 4	309,058	20.1

**Table 3:** Identification of Parkin-interacting proteins in DM platelets (below 20% coverage).

Protein ID	Protein Name	MW (Da)	% Coverage
RAC1_HUMAN	Ras-Related C3 botulinum toxin substrate 1 OS = Homo sapiens GN = RAC1 PE = 1 SV = 1	21,436	19.8
KAP0_HUMAN	cAMP-dependent protein kinase type I-alpha regulatory subunit OS = Homo sapiens GN = PRKAR1A PE = 1 SV = 1	42,955	19.4
PSA4_HUMAN	Proteasome subunit alpha type-4 OS = Homo sapiens GN = PSMA4 PE = 1 SV = 1	29,465	19.2
FA5_HUMAN	Coagulation factor V OS = Homo sapiens GN = F5 PE = 1 SV = 4	251,546	17.9
NDUAC_HUMAN	NADH dehydrogenase (ubiquinone)1 alpha subcomplex subunit 12 OS =Homo sapiensGN = NDUFA12 PE =1 SV=1	17,104	17.9
VATG1_HUMAN	V-type proton ATPase subunit G 1 OS = Homo sapiens GN = ATP6V1G1 PE = 1 SV = 3	13,749	16.9
S10A6_HUMAN	Protein S100-A6 OS = Homo sapiens GN = S100A6 PE = 1 SV = 1	10,173	16.7
EMRE_HUMAN	Essential MCU regulator, mitochondrial OS = Homo sapiens GN = SMDT1 PE = 1 SV = 1	11,434	15.9
ACO13_HUMAN	Acyl-Coenzyme A thioesterase 13 OS = Homo sapiens GN = ACOT13 PE = 1 SV = 1	14,951	15.7
UBL4A_HUMAN	Ubiquitin-Like protein 4A OS = Homo sapiens GN = UBL4A PE = 1 SV = 1	17,766	15.3
IMB1_HUMAN	Importin subunit beta-1 OS = Homo sapiens GN = KPNB1 PE = 1 SV = 2	97,108	15.1
STAT3_HUMAN	Signal transducer and activator of transcription 3 OS = Homo sapiens GN = STAT3 PE = 1 SV = 2	88,011	13.6
ATP8_HUMAN	ATP synthase protein 8 OS = Homo sapiens GN = MT-ATP8 PE = 1 SV = 1	7986	13.2
RBX1_HUMAN	E3 ubiquitin-protein ligase RBX1 OS = Homo sapiens GN = RBX1 PE = 1 SV = 1	12,266	13
THIO_HUMAN	Thioredoxin OS = Homo sapiens GN = TXN PE = 1 SV = 3	11,730	12.4
TGFB1_HUMAN	Transforming growth factor beta-1 OS = Homo sapiens GN = TGFB1 PE = 1 SV = 2	44,313	12.1
ROCK2_HUMAN	Rho-Associated protein kinase 2 OS = Homo sapiens GN = ROCK2 PE = 1 SV = 4	16,0799	12
S10A9_HUMAN	Protein S100-A9 OS = Homo sapiens GN = S100A9 PE = 1 SV = 1	13,234	11.4
QCR9_HUMAN	Cytochrome b-c1 complex subunit 9 OS = Homo sapiens GN = UQCR10 PE = 1 SV = 3	7304	11.1
PSB7_HUMAN	Proteasome subunit beta type-7 OS = Homo sapiens GN = PSMB7 PE = 1 SV = 1	29,946	10.8
TGFI1_HUMAN	Transforming growth factor beta-1-induced transcript 1 protein OS = Homo sapiens GN = TGFB1I1 PE = 1 SV = 2	49,782	10.6
GLRX1_HUMAN	Glutaredoxin-1 OS = Homo sapiens GN = GLRX PE = 1 SV = 2	11,768	10.4
ANT3_HUMAN	Antithrombin-III OS = Homo sapiens GN = SERPINC1 PE = 1 SV = 1	52,569	10.1
UB2L6_HUMAN	Ubiquitin/ISG15-Conjugating enzyme E2 L6 OS = Homo sapiens GN = UBE2L6 PE = 1 SV = 4	17,757	9.8
UFM1_HUMAN	Ubiquitin-Fold modifier 1 OS = Homo sapiens GN = UFM1 PE = 1 SV = 1	9112	9.4
MGST3_HUMAN	Microsomal glutathione S-transferase 3 OS = Homo sapiens GN = MGST3 PE = 1 SV = 1	16,506	9.2
ECHA_HUMAN	Trifunctional enzyme subunit alpha, mitochondrial OS = Homo sapiens GN = HADHA PE = 1 SV = 2	82,947	9
ECHB_HUMAN	Trifunctional enzyme subunit beta, mitochondrial OS = Homo sapiens GN = HADHB PE = 1 SV = 3	51,262	8.9
PSA5_HUMAN	Proteasome subunit alpha type-5 OS = Homo sapiens GN = PSMA5 PE = 1 SV = 3	26,394	8.7
PSA2_HUMAN	Proteasome subunit alpha type-2 OS = Homo sapiens GN = PSMA2 PE = 1 SV = 2	25,882	8.1
DNJA2_HUMAN	DnaJ homolog subfamily A member 2 OS = Homo sapiens GN = DNAJA2 PE = 1 SV = 1	45,717	8
SODC_HUMAN	Superoxide dismutase (Cu–Zn) OS = Homo sapiens GN = SOD1 PE = 1 SV = 2	15,926	7.8
UB2D2_HUMAN	Ubiquitin-Conjugating enzyme E2 D2 OS = Homo sapiens GN = UBE2D2 PE = 1 SV = 1indistinguishable	16,724	7.5
UBE2N_HUMAN	Ubiquitin-Conjugating enzyme E2 N OS = Homo sapiens GN = UBE2N PE = 1 SV = 1indistinguishable	17,127	7.2
GSTM3_HUMAN	Glutathione S-transferase Mu 3 OS = Homo sapiens GN = GSTM3 PE = 1 SV = 3	26,542	7.1
CH10_HUMAN	10 kDa heat shock protein, mitochondrial OS = Homo sapiens GN = HSPE1 PE = 1 SV = 2	10,925	6.9
UBE2O_HUMAN	(E3-independent) E2 ubiquitin-conjugating enzyme OS = Homo sapiens GN = UBE2O PE = 1 SV = 3	141,205	6.9
NDUS5_HUMAN	NADH dehydrogenase (ubiquinone) iron–sulfur protein 5 OS = Homo sapiens GN = NDUFS5 PE = 1 SV = 3	12,509	6.6
TIM16_HUMAN	Mitochondrial import inner membrane translocase subunit TIM16 OS = Homo sapiens GN = PAM16 PE = 1 SV = 2	13,816	6.4
COX5B_HUMAN	Cytochrome c oxidase subunit 5B, mitochondrial OS=Homo sapiens GN=COX5B PE=1 SV=2	13,687	6.2
CD36_HUMAN	Platelet glycoprotein 4 OS = Homo sapiens GN = CD36 PE = 1 SV = 2	53,019	5.3
COX41_HUMAN	Cytochrome c oxidase subunit 4 isoform 1, mitochondrial OS = Homo sapiens GN = COX4I1 PE = 1 SV = 1	19,564	4.7
AN32B_HUMAN	Acidic leucine-rich nuclear phosphoprotein 32 family member B OS = Homo sapiens GN = ANP32B PE = 1 SV = 1	28,770	4.4
TXND9_HUMAN	Thioredoxin domain-containing protein 9 OS = Homo sapiens GN = TXNDC9 PE = 1 SV = 2	26,517	4
ITB1_HUMAN	Integrin beta-1 OS = Homo sapiens GN = ITGB1 PE = 1 SV = 2	88,357	3.9
ROCK1_HUMAN	Rho-Associated protein kinase 1 OS = Homo sapiens GN = ROCK1 PE = 1 SV = 1	158,076	3.8
GRCR2_HUMAN	Glutaredoxin domain-containing cysteine-rich protein 2 OS = Homo sapiens GN = GRXCR2 PE = 3 SV = 1	28,266	3.6
CATA_HUMAN	Catalase OS = Homo sapiens GN = CAT PE = 1 SV = 3	59,719	3.6
VPS35_HUMAN	Vacuolar protein sorting-associated protein 35 OS = Homo sapiens GN = VPS35 PE = 1 SV = 2	91,649	3.6
ITA2B_HUMAN	Integrin alpha-IIb OS = Homo sapiens GN = ITGA2B PE = 1 SV = 3	113,306	2.9
ANGL5_HUMAN	Angiopoietin-rrelated protein 5 OS = Homo sapiens GN = ANGPTL5 PE = 2 SV = 3	44,115	2.6
FA10_HUMAN	Coagulation factor X OS = Homo sapiens GN = F10 PE = 1 SV = 2	54,697	1.4
UBP8_HUMAN	Ubiquitin carboxyl-terminal hydrolase 8 OS = Homo sapiens GN = USP8 PE = 1 SV = 1	127,444	1
UBR4_HUMAN	E3 ubiquitin-protein ligase UBR4 OS = Homo sapiens GN = UBR4 PE = 1 SV = 1	573,476	0.9
HD_HUMAN	Huntingtin OS = Homo sapiens GN = HTT PE = 1 SV = 2	347,383	0.7
MYCB2_HUMAN	E3 ubiquitin-protein ligase MYCBP2 OS = Homo sapiens GN = MYCBP2 PE = 1 SV = 3	509,759	0.6
